# Statins in Mitigating Anticancer Treatment-Related Cardiovascular Disease

**DOI:** 10.3390/ijms251810177

**Published:** 2024-09-22

**Authors:** Rong Jiang, Lian Lou, Wen Shi, Yuxiao Chen, Zhaoming Fu, Shuo Liu, Thida Sok, Zhihang Li, Xuan Zhang, Jian Yang

**Affiliations:** Department of Cardiology, The First Affiliated Hospital, Zhejiang University School of Medicine, Hangzhou 310003, China; 22318382@zju.edu.cn (R.J.); 21918203@zju.edu.cn (L.L.); 22218015@zju.edu.cn (W.S.); yuxiaochen@zju.edu.cn (Y.C.); fuzhaoming@sina.com (Z.F.); 3190105674@zju.edu.cn (S.L.); 22318952@zju.edu.cn (T.S.); 22118188@zju.edu.cn (Z.L.)

**Keywords:** chemotherapy, radiotherapy, immune checkpoint inhibitor, CAR-T cell therapy, targeted therapy, cardiotoxicity, statin

## Abstract

Certain anticancer therapies inevitably increase the risk of cardiovascular events, now the second leading cause of death among cancer patients. This underscores the critical need for developing effective drugs or regimens for cardiovascular protection. Statins possess properties such as antioxidative stress, anti-inflammatory effects, antifibrotic activity, endothelial protection, and immune modulation. These pathological processes are central to the cardiotoxicity associated with anticancer treatment. There is prospective clinical evidence confirming the protective role of statins in chemotherapy-induced cardiotoxicity. Numerous preclinical studies have demonstrated that statins can ameliorate heart and endothelial damage caused by radiotherapy, although clinical studies are scarce. In the animal models of trastuzumab-induced cardiomyopathy, statins provide protection through anti-inflammatory, antioxidant, and antifibrotic mechanisms. In animal and cell models, statins can mitigate inflammation, endothelial damage, and cardiac injury induced by immune checkpoint inhibitors. Chimeric antigen receptor (CAR)-T cell therapy-induced cardiotoxicity and immune effector cell-associated neurotoxicity syndrome are associated with uncontrolled inflammation and immune activation. Due to their anti-inflammatory and immunomodulatory effects, statins have been used to manage CAR-T cell therapy-induced immune effector cell-associated neurotoxicity syndrome in a clinical trial. However, direct evidence proving that statins can mitigate CAR-T cell therapy-induced cardiotoxicity is still lacking. This review summarizes the possible mechanisms of anticancer therapy-induced cardiotoxicity and the potential mechanisms by which statins may reduce related cardiac damage. We also discuss the current status of research on the protective effect of statins in anticancer treatment-related cardiovascular disease and provide directions for future research. Additionally, we propose further studies on using statins for the prevention of cardiovascular disease in anticancer treatment.

## 1. Introduction

Cancer survival rates have improved due to advances in cancer therapeutics [1,2]. However, oncological treatments can lead to cardiovascular damage, including heart failure (HF), hypertension, thrombosis, ischemia, and arrhythmias [3,4]. Cardiovascular diseases have now become the second most common cause of long-term illness and death among cancer survivors [2,5,6]. Traditional chemotherapy drugs like anthracyclines and cyclophosphamide can cause permanent damage to heart muscle cells, leading to acute or chronic left ventricular dysfunction (LVD), which may result in HF [7,8,9]. In a study involving older breast cancer patients, the 10-year cumulative incidence of HF was found to be 38% after receiving anthracycline-based treatments, 32.5% for those treated with non-anthracycline chemotherapy, and 29% for patients who did not undergo chemotherapy [6]. Radiotherapy can cause damage to the pericardium, myocardium, cardiac conduction system, and microvascular circulation, leading to corresponding cardiovascular diseases [4,9,10]. A comprehensive review shows that the incidence of radiation-related coronary artery disease in patients can reach up to 85% [11]. Certain tyrosine kinase inhibitors (TKIs), commonly utilized in targeted cancer therapies, have also been associated with an increased risk of heart dysfunction [4,12]. In a key clinical trial with breast cancer patients receiving trastuzumab treatment, 27% of the participants experienced some form of cardiac impairment, such as asymptomatic reductions in left ventricular systolic function, and 19% experienced HF symptoms [13]. Additionally, cardiac dysfunction resulting from cytokine release syndrome (CRS) linked to chimeric antigen receptor (CAR)-T cell therapy and myocarditis from immune checkpoint inhibitors (ICIs) are gaining attention [14]. While ICIs-related myocarditis occurs in approximately 0.5% to 1.7% of the patients, its mortality rate is notably 6 to 12 times higher than that of myocarditis unrelated to ICIs [15]. Consequently, there is an urgent need for strategies to prevent or mitigate the adverse cardiac effects of anticancer treatments.

Statins, the competitive inhibitors of 3-hydroxy-3-methyl-glutaryl-CoA reductase, were originally developed to reduce cholesterol synthesis and prevent cardiovascular diseases such as atherosclerosis [16,17]. In recent years, statins have been reported to have pleiotropic effects due to their impact on the mevalonate pathway, which is crucial for synthesizing various isoprenoids [18]. The prestigious international journal “*Cell*” has documented the anticancer and anti-inflammatory effects of statins [19,20,21,22]. Many preclinical and clinical studies have shown that statins can improve the anticancer efficacy of certain chemotherapy drugs, targeted drugs, and ICIs, and radiosensitize tumor cells and tissues [23,24,25,26,27,28,29]. The potential mechanisms behind statins’ anticancer effects include the regulation of the mevalonate pathway, autophagy, ferroptosis, and pyroptosis [30,31,32], which have been well elaborated and discussed by Jiang et al. in a review [33]. Furthermore, multiple studies have confirmed the cardioprotective properties of statins, attributable to their anti-inflammatory, antioxidative, antifibrotic, and immunomodulatory effects [33,34,35,36]. Therefore, as well-tolerated, inexpensive drugs that seemingly do not diminish the efficacy of anticancer therapy, statins are worthy of study to determine whether they can mitigate cardiotoxicity associated with anticancer therapy.

In recent years, many preclinical and clinical investigations have investigated the role of statins in chemotherapy, particularly anthracycline-associated cardiotoxicity, but fewer studies have focused on other chemotherapeutics, radiotherapy, immunotherapies, and targeted therapies. This review outlines the mechanisms underlying cardiovascular complications from anticancer therapies and examines how statins might mitigate such cardiac damage. It also summarizes current research on the protective role of statins in anticancer treatment-related cardiotoxicity and suggests directions for future studies.

## 2. The Pathogenesis of Common Cardiovascular Disease Induced by Anticancer Treatment

### 2.1. Conventional Chemotherapy and Radiotherapy

**Figure 1:**
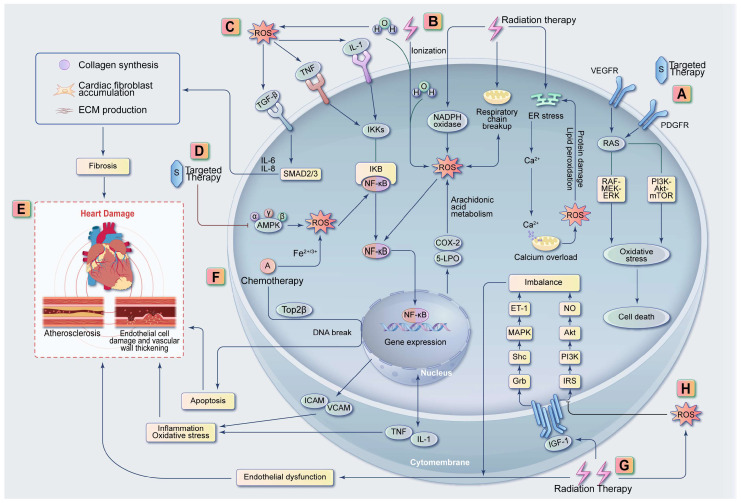
Potential mechanisms of cardiovascular damage induced by chemotherapeutics, radiotherapy, and targeted therapies. (A) Targeted therapy (e.g., sunitinib): Sunitinib induces oxidative stress and apoptosis through the Raf-MEK-ERK and PI3K-Akt-mTOR pathways. (B) Radiotherapy ionizes water molecules, damages mitochondria, induces ER stress, and activates NADPH oxidase to generate ROS. ROS further exacerbates mitochondrial damage and ER stress. (C) ROS induces inflammation through NF-κB activation, triggering fibrosis via TGF-β. (D) Sunitinib inhibits AMPK, increasing ROS production. (E) Chemotherapeutics, radiotherapy, and targeted therapies cause cardiovascular damage through mechanisms involving inflammation, oxidative stress, fibrosis, endothelial injury, and apoptosis. (F) Chemotherapeutics (e.g., anthracyclines): In the presence of iron, anthracyclines stimulate ROS production. Anthracyclines induce DNA double-strand breaks by interacting with Top2β, leading to apoptosis. (G) Radiotherapy directly damages endothelial cells and indirectly causes endothelial dysfunction through NO/ET-1 imbalance. (H) ROS causes endothelial dysfunction by inhibiting the IRS/PI3K/Akt pathway. COX-2, cyclooxygenase-2; 5-LPO, 5-lipoxygenase; ER, endoplasmic reticulum; ECM = extracellular matrix; NO, nitric oxide; PDGFR, platelet-derived growth factor receptor; ROS, reactive oxygen species; Top2β, topoisomerase II beta; VEGFR, vascular endothelial growth factor receptor.

Conventional chemotherapy and radiotherapy have been used in anticancer therapy for many years, with related cardiotoxic mechanisms being similar. Numerous reports indicate that certain chemotherapy drugs may cause cardiovascular side effects such as decreased left ventricular ejection fraction (LVEF), hemorrhagic myocarditis, pericardial effusion, myocardial infarction, HF, and arrhythmias [4,14]. The anticancer effects of radiation therapy are primarily achieved by inducing DNA damage, which leads to cell senescence and death [37]. Oxidative and nitrosative stress resulting from modifications to cellular molecules and structures also contribute to these effects. However, chest radiation therapy may expose the heart to harmful radiation, causing radiation-induced cardiovascular diseases (RICDs) like valvular dysfunction, coronary artery disease, cardiomyopathies, pericardial disease, arrhythmias, and HF [10,38]. The potential mechanisms of these cardiac side effects are as follows (Figure 1).

#### 2.1.1. ROS Generation from Anthracycline Drugs and Radiotherapy: Oxidative Stress, Endothelial Dysfunction, Inflammation, and Fibrosis

Oxidative stress arises when there is a disruption in the balance between the generation of reactive oxygen species (ROS) and the cellular antioxidant defense mechanisms. In the presence of iron, anthracyclines boost intracellular ROS production, to which myocardial cells are especially sensitive. The radiation-induced decomposition of water molecules in cells and tissues is another source of ROS [39]. Excessive ROS can damage myocardial cells through DNA damage, lipid peroxidation, and protein nitrosylation [40]. Additionally, ROS can selectively inhibit the IRS/PI3K/Akt pathway and enhance the MAPK signaling pathway. This reduces nitric oxide (NO) bioavailability, induces ET-1 production, and leads to endothelial dysfunction, inflammation, thrombosis, and eventually atherosclerosis [41,42]. ROS activates NF-κB, thereby upregulating the expression of inflammatory cytokines such as cyclooxygenase-2, 5-lipoxygenase, IL-1, and TNF, further increasing ROS production and creating a vicious cycle [43]. Additionally, NF-κB targets the genes of pro-inflammatory adhesion factors and promotes acute inflammation [44]. By activating TGF-β, ROS contributes to vascular fibrosis [45]. This increases collagen synthesis, myofibroblast accumulation, and extracellular matrix production, leading to blood vessel wall thickening and luminal narrowing [46,47,48]. Fibrosis in the conduction system can lead to arrhythmias or conduction defects. Pericardial fibrosis significantly contributes to the development of pericardial diseases [10].

#### 2.1.2. Mitochondrial Damage from Anthracyclines and Radiotherapy

The mitochondrial membrane is vulnerable to anthracycline-related cardiotoxicity. The destabilization of mitochondrial membrane potential, dissociation of the electron transport chain, elevated ROS production, and mitochondrial DNA damage likely contribute to the long-term risk of anthracycline-related cardiomyopathy [49,50,51,52]. Mitochondria, constituting a large part of cardiac myocytes and containing extranuclear DNA, are also key targets for radiation-induced cellular damage [53]. Radiation can disrupt the mitochondrial respiratory chain and impair its function, reducing ATP production and increasing ROS generation [10]. Mitochondrial dysfunction is associated with endoplasmic reticulum (ER) stress. After irradiation, the stimulated ER releases calcium ions into the cytoplasm of cardiac myocytes. This can result in mitochondrial calcium overload, triggering the release of apoptosis factors and increased mitochondrial ROS production [54].

#### 2.1.3. DNA Damage and Apoptosis from Anthracyclines and Radiotherapy

Topoisomerase II beta (Top2β) is identified as a primary target in anthracycline toxicity. The anthracycline-Top2β complex directly causes DNA double-strand breaks in myocardial cells, potentially triggering apoptosis if unrepaired [55]. A study involving mice with a cardiac-specific deletion of Top2β demonstrated a protective effect against cardiomyopathy induced by doxorubicin [55]. Radiation causes direct DNA damage, leading to strand breaks, genomic instability, and cellular apoptosis [10].

#### 2.1.4. Other Mechanisms of Cardiotoxicity of Anthracyclines

The additional mechanisms of anthracycline cardiotoxicity involve disrupting the structural sarcomeric protein, titin [56]. Reports also suggest that anthracyclines preferentially affect progenitor cells, reducing the regenerative potential of the injured myocardium [57,58,59]. Anthracyclines also damage cardiomyocytes by altering cell survival and death pathways, including those involving nuclear factor erythroid 2-related factor 2 (Nrf2). However, contradictions in these mechanisms persist, necessitating more comprehensive research for clarification. For example, doxorubicin activates Nrf2 to upregulate Hmox1 expression, leading to heme degradation and free iron release [60]. This triggers ferroptosis in cardiomyocytes [50]. Yet, Nrf2 also has antioxidant and anti-inflammatory effects in cells, potentially restoring redox homeostasis and countering doxorubicin-related cardiotoxicity [61].

#### 2.1.5. Other Mechanisms of Cardiotoxicity from Radiotherapy

Radiation inhibits the anti-inflammatory and antioxidant responses in the IRS/PI3K/Akt signaling pathway without affecting the inflammatory MAPK kinase pathway. This imbalance in NO and ET-1 production causes endothelial dysfunction, oxidative stress, thrombosis, and inflammation, ultimately contributing to the development of RICDs [62]. Consequently, this can cause myocardial ischemia and hypoxia, exacerbating myocardial damage. Micro-RNAs are involved in the development of HF resulting from cardiac radiation damage, apoptotic mechanisms, oxidative stress, inflammation, endothelial dysfunction, and fibrosis [10]. A study revealed that radiation exposure leads to increased miRNA-34a expression in the heart [63]. Radiation-induced miRNA-34a accelerates genetic aging and shortens and deactivates nuclear telomeres, leading to cardiac myocyte aging and damage [64,65]. Some miRNAs may act as biomarkers for the early detection of myocardial cytotoxicity post-radiation therapy and as potential therapeutic targets to reverse these effects [66]. Extensive research exists on miRNAs in heart diseases, yet their role in RICDs and the mechanisms through which they regulate RICDs require further investigation [67,68].

#### 2.1.6. Mechanisms of Cardiotoxicity of Other Chemotherapeutic Drugs

Other conventional chemotherapeutic agents causing cardiotoxicity include cyclophosphamide, 5-fluorouracil (5-FU), capecitabine, paclitaxel, and arsenic trioxide, though their specific mechanisms remain unclear. Cyclophosphamide can cause acute cardiomyopathy, pericardial effusion, HF, and tachyarrhythmias [69]. The metabolites of cyclophosphamide can induce endothelial capillary damage accompanied by edema, bleeding, and thrombus formation [70]. Cyclophosphamide-induced myocardial injury can lead to rapid arrhythmias and, in later stages, HF, potentially resulting in death. Lowering the dosage of this drug can significantly reduce the incidence of these adverse events. Cardiotoxicity from 5-FU and capecitabine affects up to 20–30% of the patients, varying with the study’s patient population and standards [71]. The proposed toxic mechanism involves inducing deep and diffuse vascular constriction, including in coronary microcirculation, particularly in rapidly recovering patients and those with Takotsubo syndrome [72,73]. In some patients, 5-FU may lead to vasospasm-related myocardial infarction or direct toxic damage to the myocardium and vascular system [74]. Research indicates that the direct mechanism of myocardial damage caused by 5-FU is similar to the cardiotoxic mechanism of anthracyclines, such as inducing oxidative stress in cardiomyocytes [75].

### 2.2. Targeted Therapies: Oxidative Stress, Mitochondrial Damage, and Apoptosis

Several targeted therapies, such as those directed at human epidermal growth factor receptor 2 (HER-2), MAPK/ERK kinase (MEK), vascular endothelial growth factor (VEGF), VEGF receptor (VEGFR), and BCR-ABL kinase activity, have been shown to disrupt molecular pathways that are vital for maintaining cardiovascular health. This interference can lead to cardiovascular side effects such as LVD, hypertension, cardiomyopathy, arterial thrombotic events, myocardial ischemia, arrhythmias, and HF [14,76]. The mechanisms of the cardiotoxicity of targeted drugs are different due to their different targets.

A classic example of HER-2 (also known as ERBB2) inhibitors is trastuzumab, which can lead to a decline in cardiac function or even HF in 15–45% of the patients [13,77,78]. The cardiotoxicity associated with anti-HER-2 TKIs is caused by the inhibition of the neuregulin-HER pathway (NRG-1/HER-4/HER-2 axis) [76]. Neuregulin promotes the survival and proliferation of cardiomyocytes by activating ERBB2 and ERBB4 receptors. It also maintains the normal contraction and conduction function of cardiomyocytes. Compared with normal mice, mice with heart-specific ERBB2 deficiency develop dilated cardiomyopathy and exhibit excessive contractile dysfunction under pressure overload [79]. After heart injury or myocardial infarction, the neuregulin-HER pathway can promote the regeneration and repair of cardiomyocytes. As an anti-HER-2 TKI, trastuzumab impairs the repair response of cardiomyocytes to anthracyclines, potentially leading to cardiac injury and dysfunction [9].

TKIs targeting the VEGF and MEK signaling pathways may pose the highest risk of cardiotoxicity in clinical practice. Inhibiting the VEGF pathway can impair vascular reactivity. Pre-existing or evolved coronary (micro) vascular defects may increase the risk of cardiomyopathy from VEGF inhibitors [80]. Sunitinib is a multi-target small-molecule drug targeting VEGFR. A preclinical study indicated that the inhibition of AMPK by sunitinib is linked to left ventricular systolic dysfunction (Figure 1) [81]. This inhibition results in ROS production in the mitochondria, causing mitochondrial dysfunction and cell death. Sunitinib also targets platelet-derived growth factor receptors [80]. Blocking platelet-derived growth factor receptors and VEGFR interferes with the Raf-MEK-ERK and PI3K-Akt-mTOR signaling pathways, inducing oxidative stress and triggering pro-apoptotic mechanisms such as cytochrome c release and caspase activation. These mechanisms increase cell apoptosis and impair myocardial stress response, ultimately leading to heart contractile dysfunction [76]. Nilotinib, a small molecule ABL1 inhibitor, exhibits pro-atherosclerotic properties, leading to arterial narrowing, vasospasm, and a higher risk of myocardial ischemia or infarction [82].

### 2.3. Immunotherapies

In the last decade, ICIs and CAR-T cell therapies have been widely used in clinical practice [15]. CAR-T cell therapy activates T cells using chimeric fusion proteins to recognize and target antigens present on the surface of cancer cells for destruction [83]. Small studies and case reports suggest that about 20% of the adverse events from CAR-T cell therapy are cardiovascular complications [84], commonly including arrhythmias, HF, QT prolongation, myocardial infarction, and venous thromboembolism [85,86,87]. ICIs are antibodies designed to block negative regulatory factors in T cell immune responses, relieving the inhibition of T cell activation to enhance the immune attack on cancer cells, including cytotoxic T-lymphocyte-associated protein-4, programmed cell death protein-1 (PD-1), and PD-1 ligand [88,89]. ICIs have revolutionized anticancer treatment, achieving unparalleled rates of lasting antitumor responses. However, this method can lead to systemic immune-related adverse events (irAEs) due to enhanced immune responses [90]. Myocarditis is the most frequent cardiovascular irAE. Common complications include atrioventricular conduction block, arrhythmias, and HF, which contribute to its poor prognosis [91]. Additionally, other ICIs-related cardiovascular irAEs include pericardial diseases (e.g., pericarditis and pericardial effusion), vasculitis, acute coronary syndrome, atherosclerosis-related events, thrombosis, arrhythmias, and non-inflammatory LVD [89,92,93,94,95]. Takotsubo syndrome, a specific form of LVD, is frequently reported in pharmacovigilance analyses of the patients treated with ICIs [96]. The potential pathophysiological mechanisms of these cardiovascular complications are as follows.

#### 2.3.1. CRS and Endothelial Dysfunction of CAR-T Cell Therapy

The most well-known adverse effect associated with CAR-T cell therapy is CRS, a systemic inflammatory syndrome. It is mediated by activated T cells and bystander immune cells releasing cytokines, mainly IL-6, with or without organ toxicity [97,98,99]. Generally, higher cytokine levels indicate more severe CRS. In severe CRS, the biomarkers for endothelial cell activation are elevated, including higher angiotensin-2 (Ang-2) levels in grade 4 CRS, increased Ang-2/Ang-1 ratios, and elevated von Willebrand factor (vWF) levels. Patients with severe CRS can also develop capillary leak syndrome from endothelial dysfunction [100]. In severe cases, CRS is characterized by capillary leakage, distributive shock, and multiorgan failure, which can induce significant cardiovascular strain and potentially lead to myocardial injury, negatively affecting heart function. Additionally, microvascular dysfunction and increased vascular permeability can exacerbate cardiac stress and provoke an inflammatory response in the myocardium. Procoagulant agents, such as vWF, may contribute to microvascular blockages [86]. Furthermore, when CRS compromises the integrity of the blood–brain barrier, it allows pro-inflammatory cytokines to infiltrate the cerebrospinal fluid, activating local immune and glial cells, which can result in neurotoxicity, also known as immune effector cell-associated neurotoxicity syndrome [101,102,103,104]. Additionally, titin, a protein in cardiomyocytes, is considered a putative antigen. CAR-T cells can cross-react with the heart by targeting titin, leading to cardiac damage [86].

#### 2.3.2. Abnormal Immune Responses, Inflammatory Responses and Atherosclerotic Plaque Necrosis of ICIs

The pathophysiological mechanisms of cardiovascular irAEs are not fully understood. Possible mechanisms include abnormal immune responses to cardiovascular tissues and autoimmune reactions. The post-mortem studies of two patients with ICIs-induced myocarditis revealed necrosis and the infiltration of CD4+ and CD8+ T cells, as well as macrophages, in both the myocardium and conduction system. The infiltrated myocardium, skeletal muscle, and tumors displayed similar T cell clonality [105]. This evidence suggests that T cell infiltration and inflammatory responses due to immune checkpoint inhibition might be linked to cardiovascular irAEs. Additionally, α-myosin could be a significant autoantigen in ICIs-related myocarditis, offering insights into the pathogenesis of ICIs toxicity [106]. Immune checkpoints also regulate the formation of atherosclerotic plaques [107]. The impact and mechanisms of action of ICIs on atherosclerosis are related to specific targets. Targeting PD-1 with ICIs has been reported to increase atherosclerotic plaque necrosis [108]. Conversely, targeting CD-47 with ICIs might reduce atherosclerosis progression or plaque vulnerability [109,110].

## 3. Cardiovascular Protective Effects of Statins Related to Anticancer Treatment-Induced Cardiovascular Disease

**Figure 2:**
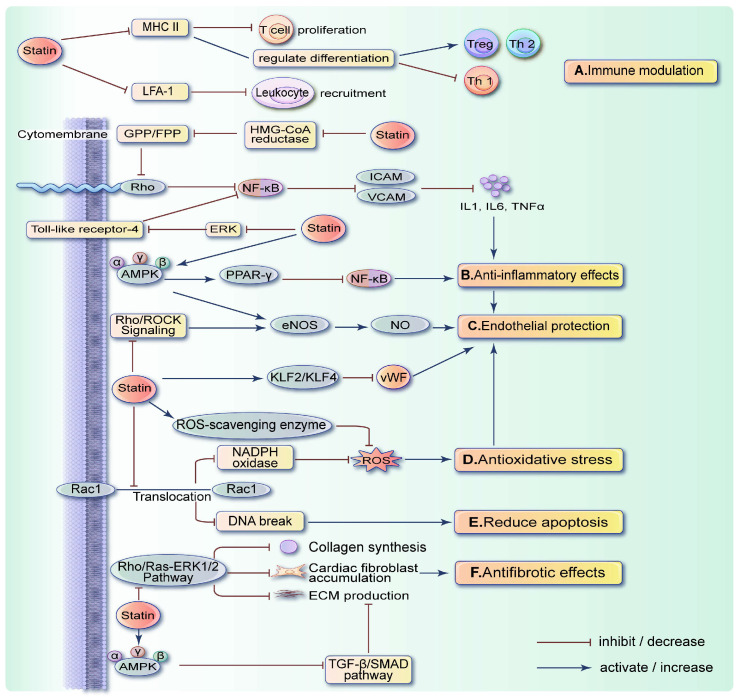
Potential mechanisms of statins that mitigate anticancer therapy-induced cardiovascular disease. (A) Statins reduce T cell proliferation, induce T cell differentiation into Th2 and Treg subsets, and decrease leukocyte recruitment, thereby exerting immunomodulatory actions. (B) Statins reduce inflammation by inhibiting NF-κB through the suppression of Rho GTPases, ERK phosphorylation, and PPARγ activation. (C) Statins protect endothelial cells by increasing NO production and reducing vWF. Their anti-inflammatory and antioxidant effects also contribute to endothelial protection. (D) Statins reduce the formation of ROS, thereby mitigating oxidative stress. (E) Statins decrease DNA damage and apoptosis by preventing Rac1 translocation to the cell membrane. (F) Statins inhibit the Rho/Ras-ERK1/2 pathway and the TGF-β/SMAD pathway, thereby exerting antifibrotic effects. MHC II, major histocompatibility complex-II; LFA-1, lymphocyte function-associated antigen-1; Treg, regulatory T cells; Th, T helper cells; GPP, geranyl diphosphate; FPP, farnesyl diphosphate; ROCK = Rho-associated protein kinase; eNOS, endothelial nitric oxide synthase; vWF, von Willebrand factor.

Originally, statins were widely prescribed to treat hyperlipidemia and prevent atherosclerotic diseases due to their lipid-lowering properties. Statins help prevent the formation and rupture of cholesterol-related plaques, as well as reduce the existing plaques and alleviate platelet-mediated vascular obstruction [111]. In recent years, studies consistently suggest that statins possess pleiotropic effects, including anti-inflammatory, antioxidative, antifibrotic, immunomodulatory, and endothelial protective properties [35,36]. These properties can reduce the harmful remodeling of heart tissue and correspond to the primary mechanisms of cardiotoxicity associated with anticancer therapies described above. These benefits do not only rely on cholesterol reduction but also on the inhibition of small GTPases such as the Ras and Rho families [112]. Small GTPases, located on the inner membrane, act as signal transducers that relay extracellular stimuli to MAPK and transcription factors, performing various functions [113] (Figure 2).

### 3.1. Antioxidant Effects and ROS Reduction

NADPH oxidase consists of enzymes Nox1-5 and Duox1-2. Regarding the antioxidant mechanism, statins downregulate Nox-1 mRNA in vascular smooth muscle cells and prevent Rac1 translocation to the cell membrane, thereby inhibiting NADPH oxidase activation and ROS production [114]. Moreover, statins have been confirmed to reduce ROS production by upregulating ROS-scavenging enzymes via S-nitrosylation, such as thioredoxin in endothelial cells [115]. Statins also exhibit antioxidant properties by activating the Keap1/Nrf2 signaling pathway [116].

### 3.2. Anti-Inflammatory Effects

The anti-inflammatory effect of statins primarily stems from inhibiting the nuclear translocation of NF-κB [117], a key inflammatory transcription factor that promotes the expression of cytokines and pro-inflammatory adhesion molecules like ICAMs and VCAMs. RhoA, Cdc42, and Rac1 are reported to stimulate the transcriptional activity of NF-κB by promoting the phosphorylation of IkappaB alpha [118]. Thus, by inhibiting Rho GTPases, statins can reduce NF-κB transcriptional activity and exert anti-inflammatory effects. The MAPK signaling pathways, including ERK, JNK, and p38, are also involved. In human umbilical vein endothelial cells (HUVECs), atorvastatin inhibits the expression of Toll-like receptor-4 mRNA by inactivating ERK phosphorylation, which indirectly hinders NF-κB activation [119]. The activation of PPARγ negatively regulates NF-κB, attenuating Ang-2-induced myocardial cell hypertrophy [120]. In macrophages, statins activate PPARγ through the RhoA-dependent signaling pathway [121]. A later study showed that simvastatin activates PPARγ and reduces NF-κB expression in the heart by inhibiting RhoA and Rho GTPase activity [122].

### 3.3. Antifibrotic Effects

In terms of antifibrosis, statins hinder the Rho/Ras-ERK1/2 pathway, decrease collagen expression, and curb fibroblast proliferation and fibrosis progression, thus mitigating myocardial hypertrophy [123]. Statins can also inhibit fibrosis by activating AMPK and downregulating the TGF-β/SMAD/CTGF pathway. The TGF-β signaling pathway induces the phosphorylation of the SMAD2/3 transcription factors. This activation prompts fibroblasts to produce the extracellular matrix, which leads to cardiac fibrosis [124]. Simultaneously, AMPK negatively regulates the TGF-β signaling pathway, thus playing an antifibrotic role [125,126].

### 3.4. Endothelial Protective Effects

The anti-inflammatory and antioxidant effects of statins can provide protection to endothelial cells. Additionally, statins protect the vascular endothelium by facilitating NO release. Rho-associated protein kinase (ROCK), a downstream target of RhoA, regulates endothelial nitric oxide synthase (eNOS) activity [127]. Statins inhibit the RhoA/ROCK signaling pathway, stabilize eNOS mRNA, and promote Akt-mediated eNOS activation, enhancing NO release [128,129]. Furthermore, statins activate AMPK, which, in turn, activates eNOS for cardiovascular protection. This mechanism has been confirmed both in vivo and in vitro [130]. Statins can reduce the expression of cytokines and matrix metalloproteinases in endothelial cells, decrease the adhesion and migration of leukocytes, and increase resistance to local injury stimuli [131]. Kruppel-like factor 2 and Kruppel-like factor 4 are also upregulated by statins [132,133]. Statins also protect endothelial cells from apoptosis and enhance the mobilization of endothelial progenitor cells [134,135].

### 3.5. Immunomodulatory Effects

The immunomodulatory effects of statins have been extensively studied in cardiovascular disease. Firstly, statins indirectly reduce the synthesis of major histocompatibility complex-II by inhibiting transcriptional regulation. This reduction in major histocompatibility complex-II expression dampens the immune response by reducing T cell proliferation and regulating T cell differentiation [36,136]. Secondly, statins limit leukocyte recruitment by interfering with adhesion mechanisms, such as lymphocyte function-associated antigen molecules, and by inhibiting chemokine secretion and ICAM-1 expression [137,138].

## 4. Use of Statins in Anticancer Treatment-Associated Cardiovascular Disease

### 4.1. Preclinical Research

Statins have been extensively studied in anthracycline-associated cardiotoxicity. Initially, using three mouse tumor models, Felezko et al. found that lovastatin potentiated the antitumor efficacy of doxorubicin while significantly reducing troponin T release from the cardiomyocytes of doxorubicin-treated mice. This study was the first to show the ability of a single drug to both improve the antitumor activity of doxorubicin and attenuate its cardiotoxicity, although other drugs also prevent doxorubicin-induced cardiotoxic effects [23]. Similarly, another study demonstrated that lovastatin reduced DNA damage resulting from the inhibition of topoisomerase II by doxorubicin through inhibiting Rac1, reducing cardiomyocyte apoptosis, without diminishing its therapeutic effects [139,140]. Other statins also provide protective effects against anthracycline-induced cardiotoxicity. Masashi et al. found that doxorubicin-induced cardiotoxicity is also attenuated by pitavastatin through its antioxidant effect involving Rac1 inhibition [141]. Alexander et al. revealed that fluvastatin increased SOD2 levels post-doxorubicin treatment, decreasing caspase-3-mediated apoptosis and cardiac inflammation [142]. In addition, atorvastatin may reduce doxorubicin-associated myocardial fibrosis and apoptosis by increasing p-Akt and decreasing p-ERK and p-JNK signaling [143]. A recent in vitro study in H9c2 cells showed that simvastatin significantly reduced doxorubicin-induced ROS, apoptosis, and cytochrome c release [144]. These studies confirm that statins have positive anti-anthracycline-associated cardiotoxic effects, involving mechanisms including antioxidation, antifibrosis, anti-inflammation, and anti-apoptosis, suggesting their potential as an adjuvant to doxorubicin. Specific molecular pathways mainly involve the inhibition of the Rho GTPase and MAPK pathways. These pleiotropic effects of statins can also mitigate heart damage from other chemotherapy drugs. For instance, simvastatin counteracts cyclophosphamide-induced cardiotoxicity by stimulating eNOS and exerting antioxidant, anti-inflammatory, and anti-apoptotic effects [145]. A study demonstrated that simvastatin prevents the disruption of the vascular constriction/dilation balance and endothelial cell function caused by 5-FU, potentially providing cardioprotective effects by regulating the ROCK/NF-κB, Akt/eNOS, and ET-1/ERK1/2 signaling pathways [146].

The anti-inflammatory, antioxidant, antifibrotic, and endothelial protection functions of statins make them theoretically appealing for treating RICDs. A study demonstrated for the first time that radiation-induced delayed injury in the lung and heart activates the Rho/ROCK and Smad pathways. In a model of radiation-induced (19 Gy) myocardial fibrosis, pravastatin showed antifibrotic effects after the activation of this pathway without interfering with previous anticancer therapy [147]. Another study showed that both pravastatin and atorvastatin maintain NO production in endothelial cells, preventing radiation-induced endothelial dysfunction. Pravastatin additionally reduces damage to mitochondrial DNA and the respiratory chain, thereby decreasing inflammatory responses [148]. Moreover, another study revealed that simvastatin (10 mg/kg/day) mitigated increases in various risk factors for cardiac disease after 10 Gy total body irradiation, including peri-arterial fibrosis, cardiac mechanical dysfunction, and the severity of myocardial infarction [149]. In one study, pretreatment with a low, physiologically relevant dose of lovastatin (1 µmol/L) protected HUVECs from radiation-induced cell death. This treatment reduced the elevation of p53/p21 protein levels and inhibited the activation of NF-κB, Chk-1, and Akt kinase following irradiation, without affecting the formation or repair of DNA double-strand breaks. However, pretreatment with 20 µmol/L of lovastatin increased radiation-induced apoptosis [150]. The effect of lovastatin on radiation-induced apoptotic cell death appears to be dose-dependent: at low doses, it functions as a radioprotective agent, while at higher doses, it acts as a radiosensitizer. In experiments with *Sprague Dawley* rats, atorvastatin reduced the expression of TGF-β1, Smad3/P-Smad3, ROCK I, and p-Akt, improving radiation-induced cardiac fibrosis, especially when atorvastatin is administered for longer periods and at higher doses (20 mg/kg/day) [151]. A recent study shows that atorvastatin can prevent left ventricular systolic dysfunction and cardiac conduction disorders after local cardiac irradiation. But the cardioprotective effects of statins observed at 30, 40, and 50 weeks vary [152]. Therefore, more animal studies are required to determine the optimal timing and dose of statin therapy.

Among the targeted drugs, the relationship between the cardiotoxicity of trastuzumab and statins is the most widely studied. In a study with 120 mice, the administration of rosuvastatin to mice treated with trastuzumab resulted in significant increases in glutathione and catalase, as well as reductions in serum CK-MB, troponin I, NT-pro BNP, TGF-β1, and IL-6 compared to the group that received trastuzumab alone, exerting antioxidant, anti-inflammatory, antifibrotic, and cardiac function protective effects. Furthermore, the study found that these effects were more pronounced when rosuvastatin was combined with ubiquinone, compared to using each drug alone [153]. Similarly, in trastuzumab and doxorubicin-induced cardiomyopathy rat models, rosuvastatin reduced systemic inflammation, as demonstrated by CRP. However, the doses of doxorubicin, trastuzumab, and rosuvastatin used in the study were much higher than the clinical doses administered to humans. Clinical studies are needed before these results can be translated to humans [154].

As for immunotherapies, there has been very little relevant research. Recently, Panagiotis et al. provided the first conclusive evidence that atorvastatin can prevent anti-PD-1-related cardiotoxicity [155]. In this research, pembrolizumab led to a broad immune-mediated inflammatory response, resulting in cardiac systolic dysfunction and microvascular coronary artery dysfunction. Atorvastatin is protective against these cardiotoxicities at both the functional and molecular levels. In EA.hy926 human endothelial cells, only high doses of atorvastatin (80 mg) attenuated pembrolizumab-induced cardiotoxicity, accompanied by decreased ICAM-1 and increased eNOS [155]. In mice, atorvastatin inhibited the release of TNF-α, IL-2, IL-6, IL-4, IFN-γ, and IL-17α, consistent with its known immunomodulatory effects, thereby preventing pembrolizumab-induced cardiac systolic dysfunction. Atorvastatin mitigated pembrolizumab-induced coronary endothelial activation and cardiac inflammation by inhibiting the increases in ICAM-1, STAT3, iNOS, and IL-6 in the myocardium. In clinical studies, atorvastatin has been proven to not interfere with the antitumor effects of pembrolizumab [25]. However, it would be better if Panagiotis et al. validated these data in a tumor-bearing in vivo model.

### 4.2. Clinical Research

In addition to preclinical studies, the ability of statins to protect against the cardiotoxicity of anticancer therapy has also been investigated in several population-based studies. In a retrospective case–control study of 628 women with breast cancer receiving anthracycline therapy, those treated with statins exhibited a lower incidence of HF compared with the matched controls [156]. Another observational study showed that statin therapy reduced the decline in LVEF caused by anthracyclines [157]. In a small-scale randomized controlled trial (RCT), the prophylactic use of atorvastatin was effective in maintaining LVEF in patients treated with anthracyclines [158]. Similarly, in an RCT involving 300 lymphoma patients receiving anthracycline-based chemotherapy, atorvastatin effectively reduced the incidence of cardiac dysfunction [159]. Based on these positive research findings, the 2022 European Society of Cardiology cardio-oncology guidelines recommend statins for primary prevention in patients at high risk of anthracycline-induced cardiotoxicity (Grade IIa, Level of Evidence B) [87]. However, a recent RCT involving 112 patients indicated that atorvastatin does not improve the decline in LVEF, ventricular remodeling, high-sensitivity troponin I, B-type natriuretic peptide, or myocardial tissue during anthracycline therapy. Nevertheless, this study has limitations, including slight imbalances between groups [160]. Recently, a retrospective analysis of 478 consecutive patients with non-small cell lung cancer (NSCLC) undergoing curative-intent (chemo)radiotherapy revealed that high-intensity statin therapy, or moderate therapy if needed, is beneficial for patients starting radiation therapy for NSCLC and may also be beneficial for those who have a sizable cardiac basal radiation dose (>3 Gy) or have pre-existing cardiovascular risk factors or have already been diagnosed with cardiovascular disease, effectively improving overall survival [161]. In contrast, a retrospective analysis of 748 patients with locally advanced NSCLC receiving thoracic radiation therapy showed that statins were inversely associated with survival rate [162]. The possible reason is that patients in the statin group have higher cardiovascular risk factors. Therefore, prospective studies are needed to explore the role of statins in radiation-induced cardiotoxicity and to provide a comprehensive evaluation of the baseline cardiac risk and cardiac radiation dose exposure. In a retrospective study of 129 HER2-positive breast cancer patients on trastuzumab treatment, Calvillo et al. revealed that the patients who also use statins experience 68% lower odds of cardiotoxicity [163]. However, another retrospective study of women aged 66 or older without prior HF, newly diagnosed with early breast cancer, showed that statin use after trastuzumab treatment did not significantly reduce the risk of HF [131]. Therefore, further research is needed in this area. As for immunotherapy, Drobni et al. reported that ICIs are linked to a high risk of aortic plaque progression and atherosclerotic cardiovascular events, such as myocardial infarction and ischemic stroke. Statin use was associated with the reduced progression of atherosclerotic plaque after ICIs [93].

## 5. Discussion

### 5.1. What’s New

This is the first comprehensive review to systematically summarize the potential protective mechanisms of statins against cardiotoxicity related to chemotherapy, radiotherapy, targeted therapy, and immunotherapy. It is the first to propose that statins may reduce CAR-T cell infiltration and myocardial damage through immunomodulation, and alleviate CAR-T cell therapy-associated cardiovascular side effects via anti-inflammatory and endothelial protective effects. In addition to covering the latest preclinical and clinical studies on statins in ICIs, radiotherapy, targeted therapy, and chemotherapy-related cardiotoxicity, the review also analyzes the current contradictions and limitations in the research. It offers several directions for future research in the following sections.

### 5.2. Limitation and Prospect

**Figure 3:**
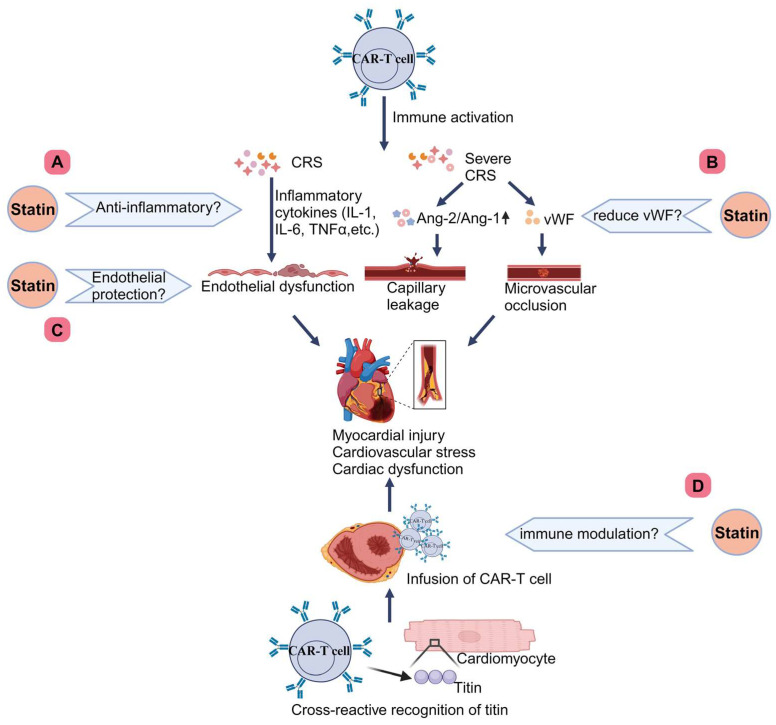
Future research directions using statins to mitigate CAR-T cell therapy-induced cardiotoxicity. (A) Investigate whether the anti-inflammatory properties of statins can reduce CAR-T cell therapy-induced inflammatory cytokines. (B) Investigate whether statins can decrease vWF levels to alleviate CAR-T cell therapy-induced microvascular obstruction. (C) Investigate whether statins can mitigate endothelial damage caused by CAR-T cell therapy. (D) Investigate whether statins can modulate the immune response to reduce CAR-T cell infiltration and subsequent myocardial injury. Created with BioRender.com. CAR, chimeric antigen receptor; CRS, cytokine release syndrome; Ang-2/Ang-1, angiotensin-2/angiotensin-1 ratio.

Table 1 records the characteristics of the preclinical and clinical studies described above. Many studies demonstrate the positive clinical translational value of statins as a protective agent against cardiotoxicity associated with anticancer therapy. However, certain issues still warrant our attention. First, more refined and in-depth basic research is required. A study showed that high doses and the prolonged use of atorvastatin could improve radiation-induced cardiac fibrosis, but another study found that the cardioprotective effect of atorvastatin was not significant at 50 weeks. Moreover, high doses of lovastatin have been shown to enhance radiation-induced apoptosis [150,151,152]. Additionally, a common side effect of statins is the potential development of myopathy, which can occur due to the depletion of energy stores in skeletal muscle, particularly at high doses of statins [164]. Therefore, future research should examine the protective effects of statins considering their type, dose, and duration. Furthermore, we recommend that the pharmacokinetics and pharmacodynamics of the drugs in the combination regimen be carefully examined in future studies. Second, the current clinical trials are limited and primarily focus on managing anthracycline-induced cardiotoxicity, with some population-based studies yielding mixed results. Well-designed, larger sample size, multicenter prospective studies are needed in the future to validate the role of statins in anticancer treatment-related cardiotoxicity. Third, no studies have applied statins to mitigate CAR-T cell therapy-associated cardiotoxicity. The current treatments for CRS caused by CAR-T cell therapy include optimal supportive care for organ toxicities, high-dose systemic corticosteroids, and anti-cytokine therapies, especially tocilizumab targeting IL-6 [165]. These treatments primarily involve immunosuppression, which can have associated toxicities. As a widely used, well-tolerated drug with immunomodulatory and endothelial protective effects, statins have been studied for CAR-T cell therapy-associated immune effector cell-associated neurotoxicity syndrome linked to CRS in a clinical trial [99]. Therefore, we also recommend investigating the protective role of statins in CAR-T cell therapy-related cardiotoxicity. Researchers can design controlled experiments to assess whether statin use reduces CAR-T cell therapy-induced inflammatory cytokines, alleviates endothelial injury, and improves microcirculatory obstruction. Additionally, in animal models, histopathological analysis can be conducted to evaluate whether T cell infiltration and associated myocardial damage are reduced (Figure 3). However, it is important to also monitor the impact of statins on the efficacy of CAR-T cell therapy and consider patient tolerance to statin treatment. Fourth, studies have shown that lovastatin and pravastatin can mitigate anthracycline- and radiotherapy-related cardiotoxicity by exerting anti-DNA damage and anti-apoptotic effects on cardiomyocytes and endothelial cells without compromising, and potentially even enhancing, anticancer efficacy [140,147]. As noted in the introduction, statins can also promote apoptosis in cancer cells and tissues, contributing to their anticancer effects. However, no study has yet explained why statins exert opposite effects on cardiac and endothelial tissues compared to cancer tissues. This needs to be elucidated in future research. Fifth, although some studies suggest that statins reduce cancer therapy-induced HF, no research has specifically explored the effects of statins on heart failure with reduced ejection fraction (HFrEF) and heart failure with preserved ejection fraction (HFpEF) separately. HFrEF has been associated with macrovascular disease and myocardial infarction, predominantly affecting men, while HFpEF is linked to microvascular dysfunction and endothelial inflammation, more commonly seen in women [166]. Due to their lipid-lowering, anti-inflammatory, and endothelial-protective properties, statins are theoretically protective in both HFpEF and HFrEF. Many studies have shown the benefits of statins for HFpEF, but two large trials have produced controversial results regarding their effects on HFrEF [167]. Moreover, evidence on gender-specific cardiac protection by statins remains limited. Future cardio-oncology research should focus on distinguishing HF subtypes related to cancer therapies, trends in cardiovascular side effects across genders, and whether statins provide differential protective effects, enabling more targeted use of statins. In addition, the impact of statins on ventricular anterior wall thickness during systole and diastole, as well as their effect on EA mitral valve velocity, may represent important areas for future research focus.

## 6. Conclusions

Due to overlapping mechanisms, statins have significant translational potential in addressing anticancer treatment-related cardiovascular diseases. Further mechanistic studies and large-scale clinical trials are needed. The efficacy of statins on various cardiovascular side effects and in different populations warrants attention. Direct evidence on the protective role of statins in CAR-T cell therapy-related cardiotoxicity remains lacking.

## Figures and Tables

**Table 1:** Studies demonstrating statins as protective agents against anticancer treatment-induced cardiovascular disease.

Study	Study Design	Anticancer Treatment	Statin	Result
Preclinical study				
Feleszko et al., Clin Cancer Res 2000 [23]	In vitro study in three murine tumor cell lines; in vivo study in three tumor models.	Doxorubicin	Lovastatin	Lovastatin both potentiated the antitumor activity of doxorubicin and reduced its cardiotoxicity.
Huelsenbeck et al., Cell Death Dis 2011 [140]	In vitro study in rat H9c2 cardiomyoblasts; in vivo study in *Balb/c* mice.	Doxorubicin	Lovastatin	Lovastatin reduced acute and subacute doxorubicin-induced heart damage and cardiac fibrosis.
Yoshida et al., J Mol Cell Cardiol 2009 [141]	In vivo study in *C57BL/6* mice and p53-deficient mice.	Doxorubicin	Pitavastatin	Doxorubicin-induced cardiotoxicity, mediated by the oxidative DNA damage-ATM p53-apoptosis pathway, was attenuated by pitavastatin through its antioxidant effect involving Rac1 inhibition.
Riad et al., Cancer Res 2009 [142]	In vivo study in 18 *C57BL/10* mice.	Doxorubicin	Fluvastatin	Pretreatment with fluvastatin attenuated doxorubicin-induced cardiotoxicity via antioxidative and anti-inflammatory effects.
Gao et al., J Cardiovasc Pharmacol 2019 [143]	In vivo study in 32 male *Wistar* rats.	Doxorubicin	Atorvastatin	Atorvastatin ameliorated doxorubicin-induced myocardial injury, fibrosis, and apoptosis.
Pecoraro et al., Int J Mol Sci 2023 [144]	In vitro study in H9c2 cells.	Doxorubicin	Simvastatin	Simvastatin co-treatment significantly reduced doxorubicin-induced cytosolic and mitochondrial ROS overproduction, apoptosis, and cytochrome c release.
Refaie et al., Hum Exp Toxicol 2022 [145]	In vivo study in 36 male *Wistar albino* rats.	Cyclophosphamide	Simvastatin	Cyclophosphamide-induced cardiotoxicity was mostly reversed by simvastatin through its antioxidant, anti-inflammatory, and anti-apoptotic actions with the stimulation of eNOS.
Muhammad et al., Sci Rep 2020 [146]	In vivo study in adult male *Wistar albino* rats.	5-FU	Simvastatin	Simvastatin normalized most of the 5-FU-induced ECG alterations, ameliorated cardiomyocyte stress and death, and improved vasoconstriction/vasodilatation equilibrium as well as endothelial cell function.
Monceau et al., Curr Drug Targets 2010 [147]	In vivo study in 150 female *C57BL/6* mice.	Radiation therapy	Pravastatin, and simvastatin	Pravastatin attenuated radiation-induced myocardial fibrosis without interfering with previous anticancer therapy.
Ait-Aissa et al., Front Cardiovasc Med 2023 [148]	In vitro study in cultured human coronary and umbilical vein endothelial cells and in vivo study in *C57BL/6* mice.	Radiation therapy	Pravastatin, and atorvastatin	Both pravastatin and atorvastatin prevented endothelial dysfunction after irradiation, with pravastatin additionally suppressing mitochondrial injury and inflammatory responses.
Lenarczyk et al., Pharmacol Res Perspect 2015 [149]	In vivo study in male *WAG/RijCmcr* rats.	Radiation therapy	Simvastatin	Simvastatin mitigated peri-arterial fibrosis, cardiac mechanical dysfunction, and the severity of myocardial infarction after irradiation.
Nübel et al., Clin Cancer Res 2006 [150]	In vitro study in HUVECs and primary human fibroblasts.	Radiation therapy	Lovastatin	Pretreatment with a low, physiologically relevant dose of lovastatin (1 µmol/L) protected HUVECs from radiation-induced cell death, whereas pretreatment with 20 µmol/L of lovastatin promoted radiation-induced apoptosis.
Zhang et al., Radiat Res 2015 [151]	In vivo study in 120 male *Sprague Dawley* rats.	Radiation therapy	Atorvastatin	Atorvastatin ameliorated radiation-induced cardiac fibrosis, particularly with longer and higher dose treatments.
Walls et al., Radiother Oncol 2024 [152]	In vivo study in 48 *C57BL/6j* mice.	Radiation therapy	Atorvastatin	Atorvastatin improved myocardial and conduction system performance after ionizing radiation in a time-dependent manner.
Kabel et al., Biomed Pharmacother 2017 [153]	In vivo study in 120 male *Balb/c* mice.	Trastuzumab	Rosuvastatin	Administration of rosuvastatin to trastuzumab-treated mice exerted antioxidant, anti-inflammatory, anti-fibrotic, and cardiac function protective effects.
Cho et al., J Am Soc Echocardiogr 2020 [154]	In vivo study in 42 female *Sprague Dawley* rats.	Trastuzumab and doxorubicin	Rosuvastatin	In doxorubicin and trastuzumab-induced cardiomyopathy rat models, rosuvastatin alleviated systemic inflammation.
Efentakis et al., Basic Res Cardiol 2024 [155]	In vivo study in *C57BL/6J* male mice and in vitro study in human EA.hy 926 endothelial cells.	ICIs (pembrolizumab)	Atorvastatin, and pravastatin	Atorvastatin protected against pembrolizumab-induced inflammatory response and mitigated coronary endothelial activation and cardiac dysfunction.
Clinical study				
Seicean et al., J Am Coll Cardiol 2012 [156]	Retrospective case–control study in 628 breast cancer patients.	Anthracycline	Statins	Incident HF and cancer-related mortality were significantly lower in the statin group.
Chotenimitkhun et al., Can J Cardiol 2015 [157]	Retrospective study in 51 patients with breast cancer, leukemia, or lymphoma.	Anthracycline-based chemotherapy	Atorvastatin, and simvastatin	Statin therapy reduced the decline in LVEF caused by anthracyclines.
Acar et al., J Am Coll Cardiol 2011 [158]	Randomized controlled trial in 40 patients.	Anthracycline-based chemotherapy	Atorvastatin	Atorvastatin was effective in maintaining LVEF in patients treated with anthracyclines.
Neilan et al., JAMA 2023 [159]	Double-blind randomized clinical trial in 300 patients with lymphoma.	Anthracycline-based chemotherapy	Atorvastatin	Atorvastatin effectively reduced the incidence of cardiac dysfunction due to anthracyclines.
Walls et al., Radiother Oncol 2023 [161]	Retrospective analysis in 478 patients with NSCLC.	(Chemo)Radiotherapy	Statins	Statin therapy effectively improved overall survival in patients with NSCLC treated with curative-intent (chemo)radiotherapy.
Calvillo-Argüelles et al., Can J Cardiol 2019 [163]	Retrospective case–control study in 525 patients with HER2+ breast cancer.	Trastuzumab-based therapy	Atorvastatin, rosuvastatin, simvastatin, and pravastatin	Statin treatment was independently associated with a lower risk of cardiotoxicity.
Drobni et al., Circulation 2020 [93]	A study situated in a single academic medical center.	ICIs	Statins	The use of statins could reduce the progression of atherosclerotic plaques after ICIs.

Abbreviations and acronyms: 5-FU, 5-fluorouracil; ECG, electrocardiogram; HUVECs, human umbilical vein endothelial cells; ICIs, immune checkpoint inhibitors; HF, heart failure; LVEF, left ventricular ejection fraction; NSCLC, non-small cell lung cancer; HER = human epidermal growth factor receptor.

## Abbreviations

HF = heart failure; LVD = left ventricular dysfunction; TKIs = tyrosine kinase inhibitors; CRS = cytokine release syndrome; CAR = chimeric antigen receptor; ICIs = immune checkpoint inhibitors; LVEF = left ventricular ejection fraction; ROS = reactive oxygen species; RICDs = radiation-induced cardiovascular diseases; COX-2 = cyclooxygenase-2; 5-LPO = 5-lipoxygenase; ER = endoplasmic reticulum; Top2β = topoisomerase II beta; Nrf2 = nuclear factor erythroid 2-related factor 2; 5-FU = 5-fluorouracil; HER-2 = human epidermal growth factor receptor 2; MEK = MAPK/ERK kinase; VEGF = vascular endothelial growth factor; VEGFR = VEGF receptor; PDGFR = platelet-derived growth factor receptor; PD-1 = programmed cell death protein-1; irAEs = immune-related adverse events; Ang-2 = angiotensin-2; Ang-2/Ang-1 = angiotensin-2/angiotensin-1 ratio; vWF = von Willebrand factor; GPP = geranyl diphosphate; FPP = farnesyl diphosphate; HUVECs = human umbilical vein endothelial cells; NO = nitric oxide; ROCK = Rho-associated protein kinase; eNOS = endothelial nitric oxide synthase; MHC-II = major histocompatibility complex-II; LFA-1 = lymphocyte function-associated antigen-1; Th = T helper cells; Treg = regulatory T cells; GPP = geranyl diphosphate; FPP = farnesyl diphosphate; RCT= randomized controlled trial, NSCLC = non-small cell lung cancer; HFrEF = heart failure with reduced ejection fraction; HFpEF = heart failure with preserved ejection fraction.
